# Factors Associated with Regional Years of Life Lost (YLLs) due to Suicide in South Korea

**DOI:** 10.3390/ijerph17144961

**Published:** 2020-07-09

**Authors:** Yoon-Sun Jung, Ki-Beom Kim, Seok-Jun Yoon

**Affiliations:** 1 Department of Public Health, Korea University, 73, Goryeodae-ro, Seongbuk-gu, Seoul 02841, Korea; sunnyaurora@nate.com (Y.-S.J.); socommat@korea.ac.kr (K.-B.K.); 2 Department of Preventive Medicine, College of Medicine, Korea University, 73, Goryeodae-ro, Seongbuk-gu, Seoul 02841, Korea

**Keywords:** suicide, years of life lost, regional difference, South Korea

## Abstract

South Korea’s suicide rate has remained among the highest in the world for some years, and there is also a gap in suicide rates among regions within the country. This study investigates the differences in years of life lost (YLLs) due to suicide in 250 districts and the factors associated with regional YLLs in South Korea in 2016. The relationships between YLLs due to suicide by region and factors such as population characteristics, health behaviors, socioeconomic factors, and mental health resources in the community were examined through multiple linear regression analysis. The gap between districts with the highest and lowest YLL due to suicide per 100,000 people was more than a 7-times difference. Factors related to YLLs due to suicide by region were physical activity rates and community mental healthcare centers, and there were differences in these factors according to gender. In conclusion, to prevent suicide at the community level, it is necessary to consider gender in establishing intervention strategies. The community needs to play an active role in promoting mental health and reducing suicide among local residents and to continue to invest in the support and management of those at high risk of suicide.

## 1. Introduction

Suicide, defined as death caused by intentional self-directed injury [1], is recognized as a critical public health issue by the World Health Organization in its Comprehensive Mental Health Action Plan [2]. According to a 2016 Global Burden of Disease (GBD) study, suicide was the leading cause of years of life lost (34.6 million) in the high-income Asia Pacific region [3]. Suicide rates have been on the decline in almost all OECD countries, but South Korea has consistently ranked near the top of the list in the past few years, with a suicide rate of 24.6 per 100,000 people in 2016 [4]. Suicide is a serious social problem in South Korea, that is, not only a public health problem at the individual level but also closely related to socioeconomic factors.

Given the seriousness and damaging nature of suicide, numerous studies have been conducted on the characteristics of and factors affecting suicide [5,6,7,8,9,10,11], but most of these studies have been conducted at the individual level. However, regional differences in suicide rates may be significant within a country; in South Korea in 2013, it was reported that the polarization of age-standardized suicide mortality rates by boroughs (*si, gun, gu*) is severe [12]. *Si, gun*, and *gu* comprise lower-level local autonomy, or municipal-level divisions, of South Korea. According to western terminology, *si* can be interpreted as a city with a population of at least 50,000, *gun* as a county with a population of less than 50,000, and *gu* as a district with a relatively smaller population. A *do*—province in western countries—is composed of *si* and *gun*, while *si* is divided into *gu* [13].

In Taiwan, another country in the Asian region, the relationship between standardized suicide rates in each of the 358 districts and socioeconomic factors by region was analyzed using spatial analysis [14]. An individual’s health status is influenced not only by personal characteristics but also by local environmental characteristics, such as the average socioeconomic level or social resources [15]. In other words, the communities where individuals reside affect their health based on the physical features of the environment, availability of health services, and other resources provided publicly or privately to support their residents, as well as socioeconomic and cultural features [16]. In South Korea, as in most countries, there are significant differences between administrative districts in demographic structure, educational and income levels, economic activity, residential environment, healthcare infrastructure, and size and composition of the local government budget, and these may be related to differences in health levels in each region. Thus, differences in health levels of local residents can be understood in terms of differences in the socioeconomic and political environments of each region.

According to research results that have revealed factors related to suicide, there are variables in which factors at the individual or regional level may be related to years of life lost (YLLs) due to suicide. Among them, a number of studies have discovered an association between suicide, the aging population, and divorce rates [17,18]. Among socioeconomic factors, the ratio of social budgets put toward mental health has been found to affect suicide rates at a regional level [19]. In addition, interventions for health behaviors, including addressing obesity and engaging in physical activity, seem to be consequential [20], as are mental health resources [18].

Disability-adjusted life years (DALYs), a single measure of population health status, are measured as the sum of years of life lost (YLLs) and years lived with disability (YLDs). YLLs, a measure of premature mortality, refers to the average length of time a person would have lived had he or she not died prematurely. As it includes the age of death rather than just the fact of its occurrence, it can be used to better quantify the burden on society from a particular cause of death [21], which can help decision-makers develop strategies to prevent premature death. In South Korea, several studies have been conducted to find out the current status of regional differences in suicide rates or related factors; however, to the best of our knowledge, regional differences in YLLs due to suicide have not been investigated.

The purpose of this study is to evaluate the differences in YLLs caused by suicide in 237 *si*, *gun*, and *gu* divisions of South Korea in 2016 and to investigate the factors related to regional YLLs.

## 2. Materials and Methods

### 2.1. Study Population

In this study, YLLs due to suicide in each basic administrative regional unit type in South Korea—*si* (city), *gun* (county), and *gu* (district)—in 2016 were defined as the study population (unit of analysis). Each of these units has its own upper-level local government (*si*, metropolitan city, or *do*, province), and each district has different health policies. A total of 237 regions were studied for YLLs due to suicide.

### 2.2. Study Design

#### Variable Selection

YLLs due to suicide was selected as the dependent variable to analyze and identify the regional differences and factors (independent variables) that account for them. Data regarding dependent variables were taken from the cause of death statistics for 2016 by the National Statistical Office. The definition of suicide in this study is death with intentional self-harm (categories X60–X89 in the database based on the ICD-10 coding system).

YLLs were calculated by subtracting the age at death from the longest life expectancy for a person of that age. We obtained data on the region, gender, and age-stratified cause-specific death from the Korean Statistical Information Service(KOSIS). When garbage codes (codes that could not be the cause of death) were input, they were distributed according to the percentage of algorithms developed in the Korean National Burden of Disease Study (KNBD) to ensure validity [22]. In order to determine possible life expectancy, standard life expectancy at each age by year and gender, based on life tables, was applied. We calculated YLLs per 100,000 population in each region to standardize YLLs according to the regional population structure.

The independent variables used in this study to identify differences in YLLs by region—*si, gun,* and *gu*—were classified into four groups: (1) population characteristics, (2) health behaviors, (3) socioeconomic factors, and (4) community resources. The details of each independent variable are summarized in Table 1.

### 2.3. Data Sources

Data sources for each of the independent and dependent variables covered in this study are summarized in Table 1. In the process of composing a dataset by combining several sources, the sources were linked based on the name of each region.

### 2.4. Statistical Analysis 

We conducted basic descriptive statistics analyzing differences in the regional burden of suicide in YLLs by *si, gun, and gu* areas. Next, multiple linear regressions were conducted to evaluate the association of each variable with regional YLLs due to suicide. To increase the confidence of the analysis results and obtain stable statistics, we tried to reduce the effects of the outliers and, ultimately, trimmed off the upper and lower 2.5th percentiles, 13 observations that were shown as extreme, in order to reduce errors (1st to 7th by low-order observations and 245th to 250th by high-order observations in each of the total, male, and female populations were removed); 237 regions remained after removal. All steps of analysis were conducted with SAS 9.4 software (SAS Institute INC., Cary, NC, USA).

## 3. Results

### 3.1. Descriptive Statistics

Descriptive statistics of the study populations are shown in Table 2. The average YLLs due to suicide over 237 regions was 501.993. YLLs due to suicide for males was 2.36 times greater than for females. The elderly population rate accounted for 17.98% of the study population, while the Korea National Statistical Office (KNSO) reported the elderly population rate in Korea as 13.2% in 2016 [29].

### 3.2. Correlations

Correlations analysis was conducted among YLLs due to suicide and regressors. The correlation between the elderly population ratio (*r* = 0.340) and YLLs due to suicide was high and statistically significant. Thus, as the elderly population ratio of a region increases, YLLs due to suicide in that region also increases. However, the divorce rate (*r* = 0.062) was rarely correlated with YLLs and was not statistically significant. As for health behaviors, physical activity rate (*r* = −0.264) was negatively correlated with YLLs due to suicide, which means that if physical activity among residents increases, YLLs due to suicide in that region decreases. Smoking rate (*r* = 0.113) and high-risk alcohol usage (*r* = 0.105) were both statistically insignificant at *p* < 0.05. In terms of socioeconomic characteristics, both financial independence rate (*r* = −0.304) and social welfare budget ratio (*r* = −0.239) were negatively correlated with YLLs and were statistically significant. For community health resources, the number of mental healthcare centers (*r* = −0.120) was correlated with YLLs due to suicide, but it failed to show statistical significance at *p* < 0.05, while the number of psychiatric clinics (*r* = −0.209) was negatively correlated with YLLs due to suicide, with statistical significance at *p* < 0.05. In terms of human-resource-related suicide, the number of psychiatrists showed a negative direction (*r* = −0.156) with statistical significance at *p* < 0.05, while the correlation result of the number of mental health nurses was not statistically significant (*r* = 0.047) at the same interval.

### 3.3. Regression Analysis

Table 3 indicates the results of regression analysis for regressors that affect YLLs due to suicide in each region. Multiple linear regression analysis was performed on a total of 237 districts. The results showed that the divorce rate had a positive association with increased YLLs, but it was not statistically significant at *p* < 0.05.

Among health behaviors, only the physical activity rate was able to explain variations in YLLs due to suicide at a significance level at *p* < 0.05 (or better). Female YLLs due to suicide had a negative association with the physical activity rate, meaning that more physical activity tended to be associated with lower YLLs due to suicide. However, male YLLs due to suicide had no association with the physical activity rate.

In terms of health-related infrastructure, the number of mental healthcare centers in the region had an association with male YLLs due to suicide, with statistical significance at *p* < 0.05. This can be interpreted as showing that more mental healthcare centers per person tend to be associated with a decreased number of suicide cases in a region. In contrast, no such statistically significant association was found for the total population or for women. In addition, there was no association found in human resource factors, namely, the number of psychiatrists and mental health nurses. Both factors showed a positive direction of coefficients according to the test result; however, it failed to attain statistical significance.

## 4. Discussion

This study investigates differences in age-standardized YLL due to suicide in 237 *si*, *gun*, and *gu* in South Korea in 2016 as well as the associated factors. As health is influenced by biomedical factors, behavioral factors, and social and physical environments through direct and indirect paths, communities and residential areas can have direct or indirect effects on health levels [30,31]. In particular, social-level suicide risk factors are not a simple summation of individual risk factors [32], so a more macroscopic approach is essential to understanding the fundamental causes of suicide.

We found that the average YLLs due to suicide by region was more than twice as high in men, (at 693,105) than in women (at 293,083). This is consistent with previous studies [33,34,35] analyzing the causes of suicide rates at the individual level.

Our results show that the YLLs due to suicide are related to regional factors such as physical activity and the number of mental healthcare centers and that these factors vary according to sex. Additionally, while the elderly population ratio, smoking rate, high-risk alcohol usage, obesity, and the number of psychiatric clinics showed positive relationships with YLLs due to suicide, and financial independence and the social welfare budget ratio showed negative relationships with YLLs due to suicide, these associations were not statistically significant in the regression analysis.

A significant finding in our study is that regional physical activity was associated with low YLLs due to suicide. This result coincides with existing studies [36], which have stated that physical activities help prevent direct suicidal behaviors as thoughts of suicide are improved by physical activity. Other studies have found that regular physical activity provides psychological benefits such as improving anxiety symptoms, reducing depression levels, and improving life satisfaction [37,38]. Regression analysis by sex found that for women, regional physical activity has an association with low YLLs due to suicide, but no such association exists for men. This may show that creating a local environment that allows residents to engage in regular physical activity could improve the psychological wellbeing of local residents, especially female residents, in the relevant areas and thereby protect them from suicide.

We also found that the presence of a community mental health welfare center was related to the suicide prevention of male residents. In Korea, mental health welfare centers are operated by public health centers or agencies commissioned by the local government, serving to establish linkages between mental health facilities in the community, in order to connect people with mental illness to communities and enable their return to society, to carry out suicide prevention activities, and to provide mental health counseling. The present results confirm the importance of such health and welfare resources at the community level. Hung and colleagues (2020) similarly stated that there were negative associations between the number of community mental health centers and suicide in the United States from 2014 to 2017, also suggesting that more access to community mental health resources can help prevent suicide [39]. Investment in suicide prevention policies from a community perspective by local governments can yield substantial results. 

South Korea is currently developing an active suicide risk identification system that will be operated from a community-centered perspective [40]. This plan focuses on analyzing an area where the suicide rate is high in terms of health and social care policies, living infrastructure, and social networking with local experts, and then establishing an area-specialized suicide prevention strategy, operating from and based on the community mental health centers and primary care services. In this respect, the present result provides evidence supporting the idea that local intervention needs to begin by expanding the accessibility of neighborhood mental health resources. In addition, our findings suggest that regional YLLs due to suicide are related to different factors according to sex, so it is necessary to prepare policies that reflect gender differences within regions rather than uniform measures.

In this study, factors found to increase the risk of suicide at the individual level in previous studies, such as the elderly population ratio, smoking rate, high-risk alcohol usage, and obesity prevalence rate, were not found to be related to regional YLLs due to suicide. This suggests that, compared to individual-level characteristics, different results are obtained when the regional characteristics of the community are considered, that is, when individual health interacts with various social and structural conditions in the region. In addition, in the correlation analysis, the financial independence of the region, its social welfare budget, and the number of psychiatric clinics also showed significant associations with regional YLLs due to suicide, but not when controlled by other variables in the multiple regression analysis. Financial independence was used as an indirect indicator, reflecting the economic level of the community, and the social welfare budget was expected to have a positive effect on the mental health of the region.

From these results, we inferred that even if the community’s financial independence is excellent or the social welfare budget is large compared to the number of residents, the local government did not actually invest in building a system or creating an environment that promotes mental health and prevents suicide among residents. In addition, mental health welfare centers, which engage in early detection and counseling for residents with mental illness and/or high risk of suicide and support case management and social return after discharge from medical institutions, were related to the reduction of suicide rates in the community, but the number of psychiatric clinics, the number of psychiatrists, and the number of mental health nurses were not. This is interpreted to mean that community-level infrastructure aimed at prevention rather than treatment or hospitalization of patients may be more effective in preventing suicide by local residents. The social welfare budget in a community should be used to solve the mental health problems of residents and manage them continuously.

There were some limitations to our study that should be reflected upon. First, the influence of the community on suicide relates to both the characteristics of the individuals who make up the community and to the factors of the community in which the individual lives. In other words, individual- and regional-level variables interact with each other to increase or decrease suicide rates in a region. However, we were unable to identify these interactions because we only used data constructed at the regional level. Second, since our study is a cross-sectional study, it was not possible to determine the relationship between regional-level factors and YLLs due to suicide. If personality characteristics are adjusted based on longitudinal data and causality between regional-level factors and suicide is identified, it will be of great help in establishing policies to prevent suicide in each region in the future. Third, overall, women had significantly lower R-squared results than men, which is similar to the results of Cheong’s study [41], which analyzed the characteristics of suicide rates at the regional level. According to Kim’s research on the characteristics of adult suicide [42], suicide in women is related to psychological problems such as depression, but our study did not consider psychological factors as a predictive factor, so the R-squared was consequently low. Additional research is needed to determine the nature of suicide by gender.

Despite these limitations, we were able to confirm that a region’s physical environment has a significant effect on regional YLLs due to suicide, and we found that these effects present differently according to sex. Above all, by using YLLs, we set a metric to measure potential life loss outside the number of deaths. By calculating the time lost based on the individual’s potential maximum life expectancy at each age, deaths of all ages contribute to quantifying the burden of premature death.

## 5. Conclusions

In conclusion, this study shows that age-standardized regional YLLs due to suicide in Korea are influenced by physical activity and community mental health welfare centers and that these factors vary according to sex. Thus, it is necessary to prepare policies considering gender, even within regions, as opposed to uniform measures to prevent suicide. The results suggest that active participation and ongoing investments to promote suicide prevention projects centered on the community can lower suicide rates in the regions.

## Figures and Tables

**Table 1:** Variable classification, definitions, and sources of years of life lost (YLLs) due to suicide (by region), population characteristics, health behavior, socioeconomic factors, and community resources.

Variables			Definition	Source
Dependent Variable	YLLs due to Suicide (by Region)		$\frac{Sum\;of\;individual\;YLLs\;due\;to\;suicide\;in\;region}{Population\;of\;region}$	Cause of death statistics from KNOS * [23]
Independent Variable	Population Characteristics	Sex ratio (%)	Number of males per 100 females	Resident registration population status data from MOIS ** (2016) [24]
		Elderly population Ratio (%)	Divorced rate per 1000 people	
		Divorce rate (%)	Percentage of population registered as over 65 years old	
	Health Behaviors	Smoking rate (%)	Percentage of population who smoke “every day” or who smoke “seldomly” but have smoked more than 100 cigarettes in their entire life	Community Health Survey from MOHW *** (2016) [25]
		High-Risk Alcohol usage (%)	The proportion of population who drank more than 5 cans of beer (for male, 3 cans for female) for at least twice a week in the last year	
		Obesity (%)	Body mass index greater than 25	
		Physical activity (%)	Walking practice rate is adopted as a proxy indicator of physical activity. The definition of walking practice is the percentage of the population who have walked more than 30 min in a day for more than 5 days in the last week	
	Socioeconomic Factors	Financial independence (%)	$Financial\;Independence\;rate\;\left(\%\right)=\frac{local\;tax\;+\;non\;-\;tax\;revenue}{general\;account\;revenue}$	Community Statistics from KNOS (2016) [26]
		Social welfare Budget ratio (%)	Proportion of local government budget scheduled for social welfare and healthcare	
	Community Resources	Numbers of psychiatric clinics	Number of psychiatric clinics per 1000 people	National Health Insurance Statistics from MOHW (2016) [27] Mental Health Status Survey from National Center for Mental Healthcare (2016) [28]
		Numbers of mental health welfare centers	Number of mental health welfare centers per 1000 people	
		Numbers of psychiatrists	Number of psychiatrists per 1000 people	
		Numbers of mental health nurses	Number of mental health nurses per 1000 people	

* Korea National Statistical Office. ** Ministry of Interiors and Safety. *** Ministry of Health and Welfare.

**Table 2:** Descriptive statistics of age- and sex-standardized YLLs due to suicide and regressors in South Korea—*si, gun, gu*.

Variables	Mean	Std. Dev.	Max.	Min.
*YLLs due to suicide (by region*)				
Total (*n* = 237)	501.993	128.110	886.620	280.978
Male	693.105	208.720	1368.71	368.834
Female	293.083	123.680	635.650	24.160
Sex ratio	100.59	5.12	132.20	91.00
Elderly population ratio	17.98	7.83	37.50	6.60
Divorce rate	2.08	0.36	3.10	1.20
Financial independence rate	23.39	14.13	61.50	4.10
Smoking rate	22.26	2.57	28.00	15.40
High-risk alcohol usage	18.59	3.41	33.10	7.40
Physical activity	39.30	11.01	69.40	17.60
Obesity	27.94	3.05	36.70	18.50
Social welfare budget rate	33.47	14.33	70.00	17.60
Numbers of psychiatric clinics per 1000 people	0.02	0.036	0.31	0
Numbers of mental healthcare centers per 1000 people	0.85	0.37	2.0	0
Numbers of psychiatrists	0.088	0.091	0.694	0
Numbers of mental health nurses	0.013	0.032	0.398	0

**Table 3:** Regression analysis of YLLs due to suicide versus regressors in 237 municipal-level divisions in South Korea, 2016 (*n* = 237).

(*n* = 237)		Total		Male		Female	
		β	Std. Error	β	Std. Error	β	**Std. Error**
Population Characteristics	Sex ratio	−1.764	1.764	−2.735	2.749	0.597	2.262
	Elderly pop. ratio	2.906	2.184	5.483	3.519	−1.328	2.262
	Divorce rate	43.822	26.640	70.735	44.135	4.101	29.798
Health Behaviors	Smoking rate	2.508	3.883	3.518	6.235	1.862	4.094
	High-risk alcohol usage	0.069	2.806	6.056	4.515	−1.607	2.948
	Obesity	1.374	3.239	5.066	5.240	−0.789	3.532
	Physical activity	−1.958 *	0.867	−0.929	1.406	−2.130 *	0.923
Socioeconomic Factors	Financial independence	−1.398	1.002	−1.516	1.637	−0.931	1.05
	Social welfare budget	0.099	0.910	0.516	1.485	1.049	0.961
dMental Health Resources	Numbers of psychiatric clinics	−171.244	288.875	−505.259	682.918	−188.222	442.298
	Numbers of mental healthcare centers	−26.418	24.459	−106.746 **	39.0460	−9.880	25.848
	Numbers of psychiatrists	109.714	160.053	154.654	270.494	144.189	173.041
	Numbers of mental health nurse	113.860	251.945	12.989	416.565	256.669	269.566
		R^2^	0.2330	R^2^	0.2142	R^2^	0.0458

* *p* < 0.05, ** *p* < 0.01.

## References

[1] Crosby A.E., Ortega L., Melanson C. (2011). Self-Directed Violence Surveillance: Uniform Definitions and Recommended Data Elements, Version 1.0.

[2] World Health Organization (2013). Comprehensive Mental Health Action Plan 2013–2020.

[3] Naghavi M. (2019). Global, regional, and national burden of suicide mortality 1990 to 2016: Systematic analysis for the Global Burden of Disease Study 2016. BMJ.

[4] Suicide Rates (Indicator).

[5] Park E. (2008). The influencing factors on suicide attempt among adolescents in South Korea. J. Korean Acad. Nurs..

[6] Kim Y., Myung W., Won H.-H., Shim S., Jeon H.J., Choi J., Carroll B.J., Kim D.K. (2015). Association between air pollution and suicide in South Korea: A nationwide study. PLoS ONE.

[7] Lee H., Hahm M., Park E. (2013). Differential association of socio-economic status with gender-and age-defined suicidal ideation among adult and elderly individuals in South Korea. Psychiatry Res..

[8] Ro J., Park J., Lee J., Jung J. (2015). Factors that affect suicidal attempt risk among Korean elderly adults: A path analysis. J. Prev. Med. Public Health.

[9] Chin Y., Lee H.Y., So E.S. (2011). Suicidal ideation and associated factors by sex in Korean adults: A population-based cross-sectional survey. Int. J. Public Health.

[10] Park S., Jang H. (2018). Correlations between suicide rates and the prevalence of suicide risk factors among Korean adolescents. Psychiatry Res..

[11] Shin K.M., Cho S.M., Hong C.H., Park K.S., Shin Y.M., Lim K.Y., Koh S.H. (2013). Suicide among the elderly and associated factors in South Korea. Aging Ment. Health.

[12] Korea National Statistical Office (2018). Cause of Death Data 2018.

[13] Korean Ministry of Interior and Safety (2019). Korean Ministry of Interior and Safety, Para 1 of Art. 7 of the Local Autonomy.

[14] Chang S.S., Sterne J.A.C., Wheeler B.W., Lu T.H., Lin J.J., Gunnell D. (2011). Geography of suicide in Taiwan: Spatial patterning and socioeconomic correlates. Health Place.

[15] Park J., Kim J., Lee K. (2018). Regional difference in preventable hospitalizations in South Korea. Int. J. Econ. Manag. Syst..

[16] Macintyre S., Ellaway A., Cummins S. (2002). Place effects on health: How can we conceptualise, operationalise and measure them?. Soc. Sci. Med..

[17] Park I.J. (2017). The effects of local government condition on the local suicide rate: A focus on the fifth local election period in South Korea. Korea. Ins. Public Affr..

[18] Yoon M.S., Choi M.M. (2012). Effects of welfare and mental health resources on the suicide in community—Focused on social welfare infra and mental health infra. J. Community Welf..

[19] Jeong J.Y., Kim C., Shin M., Ryu S.Y., Hong J., Kim N.H., Hwang T.Y., Kim H., Kim K.Y., Lee H. (2017). Factors related with regional variations of health behaviors and health status: Based on Community Health Survey and regional characteristic data. Korea Public Health Res..

[20] Lee S.Y., Heo M.R. (2015). A study on factors affecting middle-aged men’s suicidal ideation. J. Korea Acad. Ind. Coop. Soc..

[21] Gardner J.W., Sanborn J.S. (1990). Years of potential life lost (YPLL)—What does it measure?. Epidemiology.

[22] Lee Y.R., Kim Y.A., Park S.Y., Oh C.M., Kim Y.E., Oh I.H. (2016). Application of a modified garbage code algorithm to estimate cause-specific mortality and years of life lost in Korea. J. Korea Med. Sci..

[23] Korea National Statistical Office (2016). Cause of Death Data 2016.

[24] Korean Ministry of Interior and Safety (2016). Resident Registration Population Status Data 2016.

[25] Korean Ministry of Health and Welfare (2016). Community Health Survey 2016.

[26] Korea National Statistical Office (2016). Community Statistics 2016.

[27] Korean Ministry of Health and Welfare (2016). National Health Insurance Statistics 2016.

[28] Korea National Center for Mental Health (2016). Mental Health Status Survey 2016.

[29] Korea National Statistical Office (2016). Future Population Estimation Data 2016.

[30] Gehlert S., Mininger C., Cipriano-Steffens T.M., Burton L.M., Kemp S.P., Leung M., Matthews S.A., Takeuchi D.T. (2011). Placing biology in breast cancer disparities research. Communities, Neighborhoods, and Health.

[31] Marmot M., Anand S., Peter F., Sen A. (2004). Social causes of social inequalities in health. Public Health, Ethics, and Equity.

[32] Song J., Park S., Lee K., Hong H.J. (2019). Influence of area-level characteristics on the suicide rate in Korean adolescents. Psychiatry Investig..

[33] Hawton K. (2000). Sex and suicide. Gender differences in suicidal behaviour. Br. J. Psychiatry.

[34] Whitley E., Gunnell D., Dorling D., Smith G.D. (1999). Ecological study of social fragmentation, poverty, and suicide. BMJ.

[35] Qin P., Agerbo E., Westergard-Nielsen N., Eriksson T., Mortensen P.B. (2000). Gender differences in risk factors for suicide in Denmark. Br. J. Psychiatry.

[36] Cho K. (2014). Physical activity and suicide attempt of south Korean adolescents-evidence from the eight Korea youth risk behaviors web-based survey. J. Sports Sci. Med..

[37] Ekkekakis P., Backhouse S.H., Gray C.T., Lind E. (2008). Walking is popular among adults but is it pleasant? A framework for clarifying the link between walking and affect as illustrated in two studies. Psychol. Sport Exerc..

[38] Murphy M., Neville C., Neville A., Biddle S., Hardman A. (2002). Accumulating brisk walking for fitness, cardiovascular risk, and psychological health. Med. Sci. Sports Exerc..

[39] Hung P., Busch S.H., Shih Y.-W., McGregor A.J., Wang S. (2020). Changes in community mental health services availability and suicide mortality in the US: A retrospective study. BMC Psychiatry.

[40] The Office for Government Policy Coordination (2019). A National Action Plan for Suicide Prevention: Progress and Future Plans, Korea. http://www.opm.go.kr/flexer/view.do?ftype=hwp&attachNo=94247.

[41] Cheong K.S., Choi M.H., Cho B.M., Yoon T.H., Kim C.H., Kim Y.M., Hwang I.K. (2012). Suicide rate differences by sex, age, and urbanicity, and related regional factors in Korea. J. Prev. Med. Public Health.

[42] Kim H.C. (2006). A study on the characteristics of adult suicide and suicidal type. Korean J. Psychol. Soc..

